# Availability and Quality of Surveillance and Survey Data on HIV Prevalence Among Sex Workers, Men Who Have Sex With Men, People Who Inject Drugs, and Transgender Women in Low- and Middle-Income Countries: Review of Available Data (2001-2017)

**DOI:** 10.2196/21688

**Published:** 2020-11-17

**Authors:** Sonia Arias Garcia, Jia Chen, Jesus Garcia Calleja, Keith Sabin, Chinelo Ogbuanu, David Lowrance, Jinkou Zhao

**Affiliations:** 1 Joint United Nations Programme on HIV/AIDS Geneva Switzerland; 2 School of Public Health, Xiamen University Xiamen China; 3 World Health Organization Geneva Switzerland; 4 The Global Fund to Fight AIDS, Tuberculosis and Malaria Grand-Saconnex, Geneva Switzerland; 5 Center for Global Health, School of Public Health, Nanjing Medical University Nanjing China; 6 Jiangsu Provincial Center for Disease Control and Prevention Nanjing China

**Keywords:** Key populations, HIV prevalence, men who have sex with men, people who inject drugs, sex workers, transgender women, low- and middle-income countries

## Abstract

**Background:**

In 2019, 62% of new HIV infections occurred among key populations (KPs) and their sexual partners. The World Health Organization (WHO) recommends implementation of bio-behavioral surveys every 2-3 years to obtain HIV prevalence data for all KPs. However, the collection of these data is often less frequent and geographically limited.

**Objective:**

This study intended to assess the availability and quality of HIV prevalence data among sex workers (SWs), men who have sex with men (MSM), people who inject drugs, and transgender women (transwomen) in low- and middle-income countries.

**Methods:**

Data were obtained from survey reports, national reports, journal articles, and other grey literature available to the Global Fund, Joint United Nations Programme on HIV/AIDS, and WHO or from other open sources. Elements reviewed included names of subnational units, HIV prevalence, sampling method, and size. Based on geographical coverage, availability of trends over time, and recency of estimates, data were categorized by country and grouped as follows: nationally adequate, locally adequate but nationally inadequate, no recent data, no trends available, and no data.

**Results:**

Among the 123 countries assessed, 91.9% (113/123) presented at least 1 HIV prevalence data point for any KP; 78.0% (96/123) presented data for at least 2 groups; and 51.2% (63/123), for at least 3 groups. Data on all 4 groups were available for only 14.6% (18/123) of the countries. HIV prevalence data for SWs, MSM, people who inject drugs, and transwomen were available in 86.2% (106/123), 80.5% (99/123), 45.5% (56/123), and 23.6% (29/123) of the countries, respectively. Only 10.6% (13/123) of the countries presented nationally adequate data for any KP between 2001 and 2017; 6 for SWs; 2 for MSM; and 5 for people who inject drugs. Moreover, 26.8% (33/123) of the countries were categorized as locally adequate but nationally inadequate, mostly for SWs and MSM. No trend data on SWs and MSM were available for 38.2% (47/123) and 43.9% (54/123) of the countries, respectively, while no data on people who inject drugs and transwomen were available for 76.4% (94/123) and 54.5% (67/123) of the countries, respectively. An increase in the number of data points was observed for MSM and transwomen. Overall increases were noted in the number and proportions of data points, especially for MSM, people who inject drugs, and transwomen, with sample sizes exceeding 100.

**Conclusions:**

Despite general improvements in health data availability and quality, the availability of HIV prevalence data among the most vulnerable populations in low- and middle-income countries remains insufficient. Data collection should be expanded to include behavioral, clinical, and epidemiologic data through context-specific differentiated survey approaches while emphasizing data use for program improvements. Ending the HIV epidemic by 2030 is possible only if the epidemic is controlled among KPs.

## Introduction

The prevalence of infection and disease and their distribution over time are the most fundamental elements of information required to describe an epidemic and its response. The risk of transmitting HIV varies across individuals, subpopulations, and communities. The overall risk is based on a combination of prevalence levels, behaviors, and health services, and is associated with differential transmission and acquisition risks and interactions between different populations. Globally, in 2019, an estimated 62% of new HIV infections occurred among stigmatized, epidemiologically key populations (KPs) and their sexual partners [1]. KPs are defined groups who, due to specific higher risk behaviors, are at increased risk of HIV irrespective of the epidemic type or local context [2]. In addition, their behaviors are often related to legal and social issues, such as stigma. They also experience reduced access to quality and essential health services, such as pre-exposure prophylaxis and antiretroviral treatment, further increasing their HIV acquisition and transmission risk [3]. The relative risk of acquiring HIV among gay men and other men who have sex with men (MSM) is 26 times higher than that among heterosexual men. This risk is 29 and 30 times higher, respectively, for people who inject drugs compared to those who do not inject drugs and sex workers (SWs) aged 15-49 years than same-aged females who do not sell sex. Transgender women (transwomen) are 13 times more likely than adults aged 15-49 years to acquire HIV [1]. Globally, MSM, people who inject drugs, and SWs accounted for 23%, 10%, and 8% of all new HIV infections in 2019 [1].

The geographic distribution of risks for acquiring and transmitting HIV is heterogeneous. Injection drug use and sex work occur more frequently in urban areas or areas with better economic development opportunities, though they are not absent from rural areas [4,5]. In many countries, MSM and transwomen migrate to urban areas in an attempt to leave behind repressive social stigma and discrimination [6]. Thus, spatial distribution of risks and HIV prevalence is complex across and within countries, and geographic mobility poses a unique challenge to health service continuities. It is impractical to implement surveillance and survey activities that can be used to generate prevalence estimates for all relevant subnational units (SNUs) within a country. However, for HIV prevention, care and treatment programming must be implemented efficiently so as to achieve a population-level impact, and a good understanding of the distribution of HIV infections among and across communities is required. The absence of HIV prevalence data among a specific KP is often the result of either the lack of appreciation that a population is affected differently at the local level due to its risk profile, or the refusal to recognize the existence of a specific KP. The omission of a surveillance approach that includes information on HIV prevalence among KPs can be attributed to structural stigma and discrimination toward these populations [7]. Latent or blatant structural stigma and discrimination toward KPs obstruct progress in the HIV response, including the 95-95-95 targets and impact goals. The behavioral characteristics that define each KP may not have been formally recognized at one time or another in all countries [8].

In many countries, HIV prevalence data have been collected among KPs since the late 1980s. Typically, HIV sentinel surveys were established in cities. Sentinel survey activities have continued in some countries and locales without pause over the ensuing 30 years, while others have stopped for various reasons [9]. Today, more than 120 countries report routine sentinel surveillance or surveys among 1 or more KPs. The number and periodicity of surveys over time, preferably using consistent methods and producing data amenable to analysis, can vary widely. Trend analysis, in particular, must consider the periodicity and recency of available data as well as the number or proportion of SNUs from which the data have been generated.

Although the World Health Organization (WHO) guidelines [10] recommend that bio-behavioral surveys (BBS) be implemented every 2-3 years in all relevant KPs and in all appropriate geographic areas within a country, in practice, many countries conduct BBS periodically, as resources allow. Irrespective of whether the country conducts sentinel surveillance surveys or more intensive BBS, even the most complete models include at least 1 site in the capital of each province. However, almost no data exist beyond the provincial capitals. Furthermore, some countries rely on sporadically collected data and, at times, from only 1 city. These data, regardless of the often-limited number and distribution of SNUs from which they have been generated, are frequently used as the basis for national estimates and a planning resource for a country’s national epidemic response. In each case, assumptions must be made about the distribution of HIV infections, and some form of extrapolation is necessary to estimate the burden of HIV among KPs at the national level.

Another important consideration involves the quality of individual survey results used for generating HIV estimates. A good quality survey will provide valid estimates that are statistically representative of the population being surveyed. Representativeness is a challenge for surveys among KPs due to the absence of a sampling frame [10]. Sentinel surveillance often involved convenience sampling in the 1990s and early 2000s. In such cases, the methodological consistency and trend analysis of HIV prevalence outweigh the representativeness. More recent surveys applied approximate probability sampling, such as respondent-driven sampling (RDS) and time-location sampling (TLS) [11].

Some efforts have been made to assess HIV prevalence data with similar parameters, such as KP groups, survey sites, HIV prevalence, sample size, and sampling method, among KPs at the regional level [12-18]. Most addressed a specific region or group, or covered a shorter period. Additionally, systematic assessment methods were not reported. The present analysis provides a complete and extensive review of the HIV prevalence studies/reports among KPs from 2001 to 2017. We describe the availability and quality of HIV prevalence data among KPs in 123 low- and middle-income countries, which account for more than 90% of the estimated global population living with HIV in 2019 [1]. Similar assessment methods for the availability and quality of population size estimates were published in 2016 [19].

## Methods

### Data Sources and Elements

We examined a variety of health research sources for published and unpublished literature that included HIV prevalence data for each of the 4 aforementioned KPs, namely MSM, SWs, people who inject drugs, and transwomen.

The following data sources were examined, irrespective of their language: Country Progress Reports for the United Nations General Assembly Special Session on AIDS; Global AIDS Response Progress Reports or Global AIDS Monitoring reports submitted to the Joint United Nations Programme on HIV/AIDS (UNAIDS); AIDS Data Hub [20]; annexes to Global Fund concept notes, funding requests, and programmatic gap analysis tables contained therein; and publicly available documents, including integrated BBS or sentinel surveillance reports; journal articles available on PubMed; grey literature available on websites; and technical meeting abstracts, such as those at International AIDS Conference sites. The terms used to search the publicly available documents included “key populations,” “most at-risk populations,” “risk groups,” “men who have sex with men,” “gay,” “gay men,” “sex workers,” “female sex workers,” “male sex workers,” “transgender sex workers,” “transgender,” “transgender women,” “transwomen,” “injecting drug users,” “injection drug users,” “drug users,” and “people who inject drugs.” Acronyms, including “MSM,” “FSW,” “MSW,” “TG,” “PWID,” and “IDU,” and their variations in different languages were also utilized. The main languages used in the search were English, Spanish, French, Portuguese, Russian, and Chinese. In addition, we abstracted data from reports in Arabic, Nepali, and Farsi. The assessment was limited to 123 countries that were eligible for funding from the Global Fund after the introduction of its new funding model in 2014 [21].

The reports were obtained either directly from UNAIDS or the Global Fund. The obtained reports and articles were utilized in the following priority order: (1) Reports submitted to UNAIDS or the Global Fund by funded countries, either in a draft or the final format, (2) journal articles, and (3) other grey literature available online. In case an article was based on a submitted report, we obtained the information from the report.

UNAIDS, WHO, and the Global Fund utilize a common systematic mapping approach based on abstraction of standard metadata from each survey report from 2001 onwards. This approach was used on all surveys conducted between 2001 and 2017 to conduct a basic quality assessment, and it included the name of the site, HIV prevalence, sampling method, and sample size. The survey or sentinel surveillance had to have been conducted between 2001 and 2017 for the data to be included in the assessment. The year in which the survey or sentinel surveillance was completed was used. When the survey or sentinel surveillance completion date was not available, the publication date was used as a proxy. The first-order of subnational administrative units (SNU1), available from the World Factbook [22], such as province, region, or state, were used to compare the distribution of the survey or sentinel surveillance sites to define the geographic coverage.

An initial review/analysis of all the studies meeting the aforementioned criteria was conducted in December 2016 and later updated in July 2018.

### Categorization and Scoring

**Figure 1:**
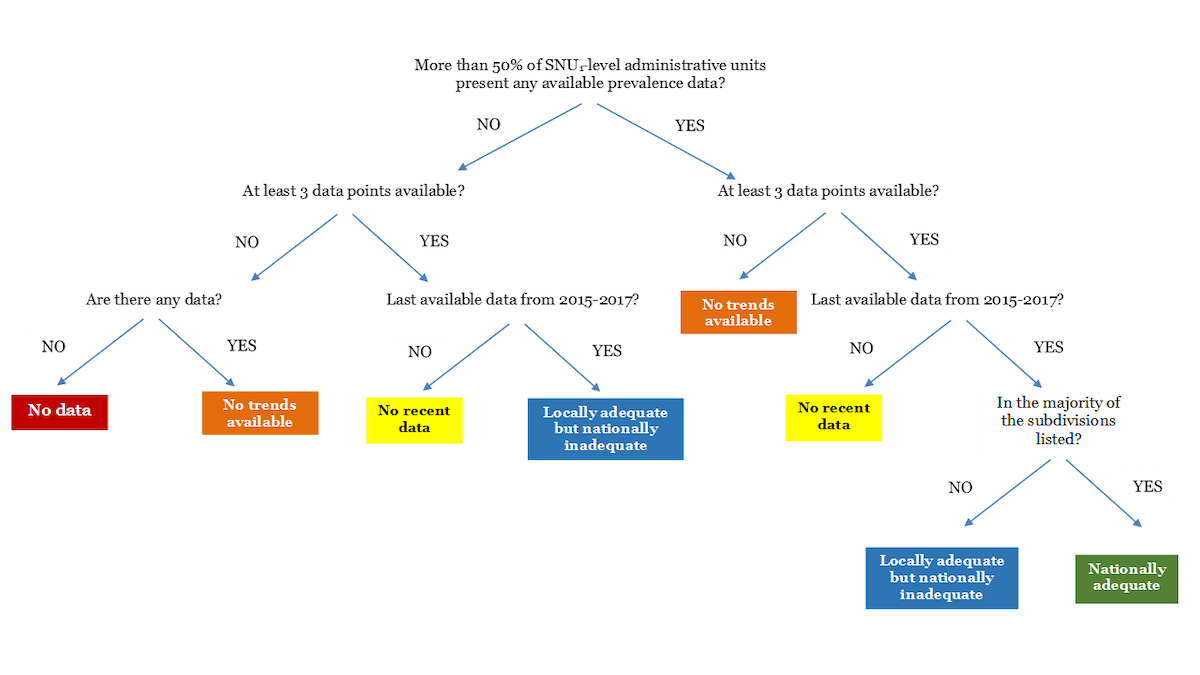
Decision tree depicting categorization of the subnational HIV prevalence data by key population and country. SNU_1_: first-order subnational administrative unit, such as a province, state, or region.

All the HIV prevalence data were categorized by country and KP group regarding availability and quality in descending order from (1) to (5) according to criteria that considered the geographical coverage, availability of trends over time, and recency of estimates (Figure 1).

(1) Nationally adequate: This category comprised countries with more than 50% of SNU_1_-level administrative units presenting any available prevalence data, with most having at least 3 data points, the last one between 2015 and 2017.

(2) Locally adequate within selected SNUs but nationally inadequate: This category comprised two scenarios, namely

(a) countries with less than 50% of SNU_1_-level administrative units with any data, with the majority having at least 3 data points, the last one between 2015 and 2017, or

(b) countries with more than 50% of SNU_1_-level administrative units with any data, with the majority not meeting the criteria of having at least 3 data points, the last one between 2015 and 2017.

(3) No recent data: This category comprised countries with at least 3 available data points for one or more of the SNUs listed, but the last data point was collected prior to 2015.

(4) No trends available: This category comprised countries with less than 3 available data points for any SNU.

(5) No data: This category comprised countries with no HIV prevalence data for any KPs since 2001.

Quality was further assessed in terms of sampling methods and sample size. Sampling methods were classified as probability (eg, simple random, systematic, or stratified sampling), approximate probability (eg, RDS or TLS), and nonprobability (eg, snowball) methods. Sample size data were grouped as <100 or ≥100. For any SNU (SNU_1_ or otherwise) with 3 or more data points for any KP group, the sampling methods were examined to assess whether they were consistent over time.

Maps were generated using Q-GIS (version 3.8 ; QGIS Development Team) [23]. Regional divisions for UNAIDS were used, while country-specific shapefiles were downloaded from GADM, a database of the location of the world’s administrative areas.

## Results

**Figure 2:**
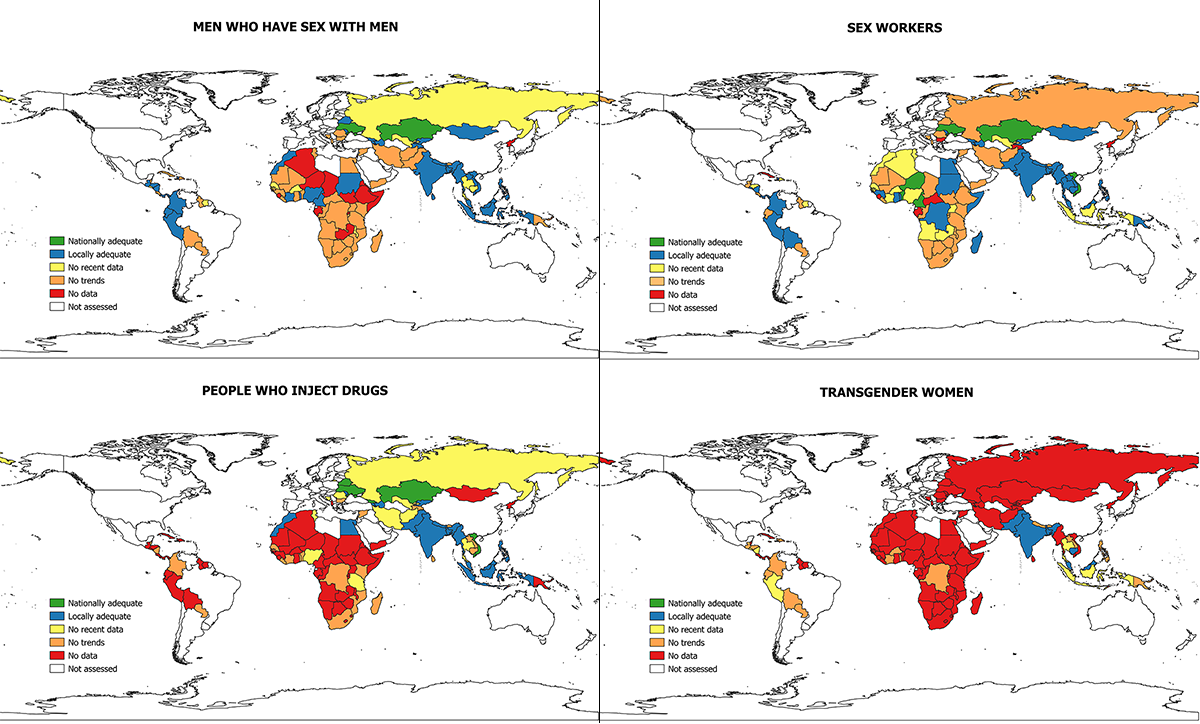
HIV prevalence categorization by key population (2001-2017). Kosovo and Zanzibar are not included due to unavailability of their shapefiles.

Of the 123 countries (for the full list, see Multimedia Appendix 1) assessed, 113 (91.9%) had at least 1 data point/prevalence estimate for at least 1 KP group during the period of 2001-2017. During the same period, 10 countries (Dominica, Gabon, Grenada, Democratic People’s Republic of Korea, Marshall Islands, Saint Lucia, Samoa, Sao Tome and Principe, Sierra Leone, and Tuvalu) did not report any HIV prevalence data for any KP group (Figure 2).

Among the 123 countries assessed, prevalence data for HIV were available for SWs in 86.2% (106/123); MSM in 80.5% (99/123); people who inject drugs in 45.5% (56/123); and transwomen in 23.6% (29/123) of the countries. KP group-specific categorization results are presented by country in Figure 2. Approximately half of the countries assessed (63/123, 51.2%) presented data for at least 3 KPs. Very few countries (13/123, 10.6% in all; 6/123, 4.9% for SWs; 2/123, 1.6% for MSM; and 5/123, 4.1% for people who inject drugs; Table 1) were categorized as having “nationally adequate” data for any single KP. Further, while 26.8% (33/123) countries were categorized as having data that were “locally adequate” within the selected SNUs, they were considered as “nationally inadequate”, primarily for SWs and MSM.

Of the 123 countries assessed, 78.0% (96/123) had data for at least 2 groups; and 51.2% (63/123), for at least 3 groups. Only 14.6% (18/123) had data for all 4 groups (Table 2).

Among SWs and MSM, “no trends available” was the most common category, followed by “locally adequate but nationally inadequate.” For people who inject drugs and transwomen, the most common category was “no data,” followed by “no trends” (Table 1).

**Table 1:** Categorization of HIV prevalence data by KP^a^ and region (2001-2017).

KPs				Regions							
				Asia and Pacific (n^b^=28)	Eastern and Southern Africa (n^b^=22)	Eastern Europe and Central Asia (n^b^=16)	Latin America (n^b^=22)	Middle East and North Africa (n^b^=11)	Western and Central Africa (n^b^=24)	Total (N^c^=123)	
	**SWs^d^, n (%)**										
		Nationally adequate	1 (3.6)		0 (0)	2 (12.5)	0 (0)	0 (0)	3 (12.5)	6 (4.9)	
		Locally adequate but nationally inadequate	13 (46.4)		1 (4.5)	5 (31.3)	7 (31.8)	3 (27.3)	4 (16.7)	33 (26.8)	
		No recent data	2 (7.1)		4 (18.2)	2 (12.5)	3 (13.6)	4 (36.4)	5 (20.8)	20 (16.3)	
		No trends	7 (25.0)		17 (77.3)	5 (31.3)	7 (31.8)	3 (27.3)	8 (33.3)	47 (38.2)	
		No data	5 (17.9)		0 (0)	2 (12.5)	5 (22.7)	1 (9.1)	4 (16.7)	17 (13.8)	
	**MSM^e^, n (%)**										
		Nationally adequate	0 (0)		0 (0)	2 (12.5)	0 (0)	0 (0)	0 (0)	2 (1.6)	
		Locally adequate but nationally inadequate	12 (42.9)		0 (0)	7 (43.8)	8 (36.4)	2 (18.2)	4 (16.7)	33 (26.8)	
		No recent data	2 (7.1)		1 (4.5)	3 (18.8)	2 (9.1)	0 (0)	2 (8.3)	10 (8.1)	
		No trends	8 (28.6)		15 (68.2)	4 (25.0)	9 (40.9)	5 (45.5)	13 (54.2)	54 (43.9)	
		No data	6 (21.4)		6 (27.3)	0 (0)	3 (13.6)	4 (36.4)	5 (20.8)	24 (19.5)	
	**People who inject drugs, n (%)**										
		Nationally adequate	1 (3.6)		0 (0)	4 (25.0)	0 (0)	0 (0)	0 (0)	5 (4.1)	
		Locally adequate but nationally inadequate	8 (28.6)		0 (0)	4 (25.0)	0 (0)	2 (18.2)	0 (0)	14 (11.4)	
		No recent data	2 (7.1)		2 (9.1)	5 (31.3)	0 (0)	2 (18.2)	1 (4.2)	12 (9.8)	
		No trends	5 (17.9)		6 (27.3)	2 (12.5)	4 (18.2)	2 (18.2)	6 (25.0)	25 (20.3)	
		No data	12 (42.9)		14 (63.6)	1 (6.3)	18 (81.8)	5 (45.5)	17 (70.8)	67 (54.5)	
	**Transwomen, n (%)**										
		Nationally adequate	0 (0)		0 (0)	0 (0)	0 (0)	0 (0)	0 (0)	0 (0)	
		Locally adequate but nationally inadequate	5 (17.9)		0 (0)	0 (0)	0 (0)	0 (0)	0 (0)	5 (4.1)	
		No recent data	2 (7.1)		0 (0)	0 (0)	3 (13.6)	0 (0)	0 (0)	5 (4.1)	
		No trends	6 (21.4)		1 (4.5)	0 (0)	8 (36.4)	0 (0)	4 (16.7)	19 (15.4)	
		No data	15 (53.6)		21 (95.5)	16 (100.0)	11 (50.0)	11 (100.0)	20 (83.3)	94 (76.4)	

^a^KP: key population.

^b^Number of countries in a region.

^c^Total number of countries assessed.

^d^SWs: sex workers.

^e^MSM: men who have sex with men.

**Table 2:** Number and proportion of countries distributed by number of KPs^a^ with HIV prevalence SNU^b^ data (2001-2017; N=123).

Status of HIV prevalence SNU data	Number of countries, n (%)
No data	10 (8.1)
1 population	17 (13.8)
2 populations	33 (26.8)
3 populations	45 (36.6)
4 populations	18 (14.6)

^a^KP: key population.

^b^SNU: subnational unit.

Different patterns of availability and quality were evident among the regions. Considering the top 2 categories, namely “nationally adequate” and “locally adequate,” on average, availability and quality of data for SWs were better in the Asia and Pacific region than in other regions. Better data availability and quality were observed for MSM in the Asia and Pacific and Eastern Europe and Central Asia regions. While data for people who inject drugs were available for countries in the Eastern Europe and Central Asia regions, the majority were categorized as “no recent data.” Most countries in the other regions were categorized under “no data” for people who inject drugs.

Multimedia Appendix 2 presents the number of HIV prevalence data points available by KPs over time as well as the availability of sample size information and number of data points with a sample size>100. From 2001 to 2017, the number of data points increased with fluctuations for MSM and transwomen, fluctuated but stabilized for people who inject drugs, and fluctuated but decreased for SWs. Information on sample size was available for more than 80% of the HIV prevalence data points for MSM (1217/1456, 83.6%) and transwomen (226/273, 82.8%), and for more than 50% of the HIV prevalence data points for SWs (2470/4368, 56.5%) and people who inject drugs (1194/2211, 54.0%). Among those data points with reported sample sizes, 83.9% (1002/1194) reported a sample size>100 for people who inject drugs; and 82.3% (1002/1217), for MSM. An overall increase in the number and proportions of data points with sample sizes>100 was observed, especially for MSM, people who inject drugs, and transwomen.

Table 3 presents the sampling methods used for surveys or sentinel surveillance in those SNUs with at least 3 data points for any KP (n=68). Of the total SNUs, 9.5% (416/4368), 19.2% (280/1456), 11.3% (249/2211), and 20.9% (57/273) met the criteria of 3 data points per KP for SWs, MSM, people who inject drugs, and transwomen. Overall, a small proportion of SNUs, namely 18.3% (76/416) for SWs, 10.7% (30/280) for MSM, 10.4% (26/249) for people who inject drugs, and 24.6% (14/57) for transwomen, used probability sampling or approximate probability sampling. Snowball sampling was the most common nonprobability sampling method across all KP groups. Moreover, 46.6% (194/416) of those SNUs reporting at least 3 data points for SWs did not report any sampling methods. Cluster, systematic, simple random, or stratified sampling methods were mostly applied from 2001 to 2008 and in a few countries where venue- or site-based sentinel surveillance had been implemented (data not shown). The comparable proportions for the other KPs are as follows: 35.4% (99/280) for MSM, 39.8% (99/249) for people who inject drugs, and 29.8% (17/57) for transwomen (Table 3).

**Table 3:** Sampling methods used for surveys in SNUs^a^ with at least 3 data points for any KP^b^ (N^c^=68).

Sampling methods				KPs				
					SWs^d^ (N^e^=416)	MSM^f^ (N^e^=280)	People who inject drugs (N^e^=249)	Transwomen (N^e^=57)
	**Nonprobability sampling, n (%)**							
			Cluster		34 (8.2)	10 (3.6)	17 (6.8)	5 (8.8)
			Convenience		32 (7.7)	18 (6.4)	10 (4.0)	2 (3.5)
			Facility-based		15 (3.6)	13 (4.6)	15 (6.0)	6 (10.5)
			Snowball		62 (14.9)	99 (35.4)	74 (29.7)	13 (22.8)
			Other nonprobability sampling		3 (0.7)	11 (3.9)	8 (3.2)	0 (0)
	**Probability sampling, n (%)**							
		Proportionate to population size			12 (2.9)	5 (1.8)	3 (1.2)	2 (3.5)
		Respondent-driven sampling			9 (2.2)	4 (1.4)	4 (1.6)	3 (5.3)
		Systematic, simple random, or stratified			41 (9.9)	14 (5.0)	12 (4.8)	7 (12.3)
		Time-location sampling			14 (3.4)	7 (2.5)	7 (2.8)	2 (3.5)
	**Unknown, n (%)**				194 (46.6)	99 (35.4)	99 (39.8)	17 (29.8)

^a^SNU: Subnational unit.

^b^KP: key population.

^c^Total number of countries.

^d^SWs: sex workers.

^e^Total number of SNUs with at least 3 data points.

^f^MSM: men who have sex with men.

We also examined the sampling methods used at the country level. In terms of KPs, the results showed that 47.2% (58/123), 36.6% (45/123), 25.2% (31/123), and 8.1% (10/123) of the countries reported sampling methods for at least 3 data points for MSM, SWs, people who inject drugs, and transwomen, respectively. Among these groups of countries, 12.2% (15/123), 12.2% (15/123), 6.5% (8/123), and 1.6% (2/123) of the countries used approximate probability sampling methods consistently for MSM, SWs, people who inject drugs, and transwomen, respectively (data not shown).

Of the 106 countries that presented any subnational HIV prevalence data among SWs, 14.2% (15/106; Bangladesh, Nepal, Pakistan, Papua New Guinea, Philippines, Thailand, Paraguay, Peru, Guyana, Suriname, Egypt, Bolivia, Guatemala, Paraguay, and Côte d'Ivoire) had disaggregated data for male SWs. Of the 56 countries that presented any prevalence data for people who inject drugs, 16.1% (9/56) (Bangladesh, Nepal, Philippines, Kenya, United Republic of Tanzania, Republic of Moldova, Russia, Egypt, and Iran) had disaggregated data for female injectors. Moreover, 26.8% (33/123) of countries (Belize, Bangladesh, Bhutan, Bolivia, Cambodia, Columbia, Costa Rica, Cote d’Ivoire, Dominica Republic, Democratic Republic of Congo, Ecuador, El Salvador, Fiji, Guatemala, Guyana, Haiti, Honduras, India, Indonesia, Laos, Malaysia, Nepal, Nicaragua, Panama, Paraguay, Peru, Philippines, Papua New Guinea, Samoa, Thailand, Tuvalu, Uruguay, and Vanuatu) clearly listed transwomen as the target population for the survey at least once; note that transwomen may have been included in the surveys on MSM in other countries.

## Discussion

Despite general improvements in the availability and quality of HIV prevalence estimates among KPs in low-and middle-income countries from 2001 to 2017, both remain woefully inadequate compared with HIV surveillance data from general populations (ie, antenatal care (ANC) sentinel surveillance and population-based estimates of HIV prevalence). These essential epidemiologic data are critical for monitoring the HIV epidemic and response regardless of epidemic type. Very few assessed countries (13/123, 10.6%) were categorized as having “nationally adequate” data for any single KP. Only 26.8% (33/123) of countries assessed were categorized as having data that were “locally adequate within selected SNUs but nationally inadequate,” primarily for SWs and MSM. In total, only 37.4% (46/123) of countries assessed had at least locally adequate data for local program planning and monitoring. Lack of nationally representative data presents a great challenge for strategic planning, advocacy, and target setting, as well as cascade analyses, which allow programs to identify and target differential access, coverage, and quality of services. Countries may misuse subnational data from a small proportion of the population to represent the entire country without appropriate adjustment. Such misuse of data can lead to incorrect resource allocation and overall estimation of HIV burden. Models, such as those used in the Spectrum suite (Avenir Health), provide one approach to extrapolate data, though input data quality must still be adequate.

Global, regional and national HIV prevalence trends are published by UNAIDS annually. However, tracking the trends of HIV prevalence among KPs remains a major challenge. Even till 2017, 36 years into the HIV epidemic, many programs still could not assess whether the programmatic responses among KP communities were improving or regressing. In Africa, where the HIV epidemic is more generalized, epidemics among the KPs were mostly neglected until very recently, when these countries started to gain epidemic control in the general population. About half (59/123, 48.0%) and one-third (46/123, 37.4%) of countries assessed presented at least 3 data points for SWs and MSM, respectively, from 2001 to 2017, despite the technical guidance urging the collection of such data every 2-3 years [10,24,25]. This challenge is the greatest in Eastern Europe and Central Asia for all 4 groups, followed by Latin America and the Caribbean, and the West and Central Africa regions.

Statistically representative estimates require the use of a probability sampling method and an adequate sample size. The use of approximate probability sampling methods has increased since 2003, especially in the last 5 years for MSM and SWs (data not shown). However, consistent use of such sampling methods remains limited. Inadequate sample sizes remain an issue despite an increasing trend in the number of estimates (among MSM, people who inject drugs, and transwomen) and surveys reporting sample size information (among SWs and people who inject drugs) (Multimedia Appendix 2).

Collecting information on the HIV epidemic among KPs continues to be uniquely challenging. While HIV surveillance approaches within generalized epidemics continue to shift toward reliance on routine ANC programmatic testing data, KP programmatic data face several important hurdles. The first hurdle concerns KP group disaggregation. In order to be able to disaggregate HIV testing data by KP groups, it would be necessary to collect and report information on these groups. The second issue concerns coverage and representativeness; whereas high majorities of pregnant women attend first ANC visits, HIV testing service coverage among KPs is more variable and difficult to define. Service providers need to be properly sensitized and trained for risk ascertainment and documentation. However, any activities that could potentially unmask people who engage in stigmatized or illegal behaviors should be avoided, or at least carefully balanced against the rights and safety of community members, who should be closely involved with related policy and technical decisions. Such efforts should continue to ensure that essential health data are also collected directly from within the communities, including through community-led monitoring, and settings that provide prevention, care, and treatment services to these populations. Such approaches will enable evolution toward more integrated and sustainable approaches, ensuring strategic information in the long term.

Health information systems must be strengthened to enable collection of person-centered individual-level longitudinal information about KPs. This strengthening should include investments in robust data security and confidentiality procedures. It is important to establish shared ownership and integration of health information systems between government institutions and nongovernmental organizations (NGOs), with involvement of communities and civil society playing an essential part. As a first step, systems that collect prevention and HIV testing data for KPs (usually owned by NGOs, at physical or virtual locations) should be linked with systems that collect treatment data (typically owned by governmental institutions). Using common unique identification standards, the entire prevention, testing, treatment, and viral load cascades can be measured for the populations served at any given point in time. Again, it is important to emphasize that data security concerns may prevent NGOs from sharing individual-level data [26], and this aspect must be adequately addressed to alleviate community concerns. Some examples indicate how HIV-related data among KPs are gathered from all service providers in a single information system [8]. Such an arrangement will ensure that programmatic and real-time data can be used for monitoring the epidemic among KPs. While external donors shall continue financing such data systems and building in-country capacity, national stakeholders need to increase financing for government institutions and NGOs for long-term, localized solutions for collection and use of quality data.

There are several limitations to this analysis. This analysis did not include data collected from prisoners and some other affected populations defined in the WHO guidelines [2]. Despite the efforts to collect and review all survey data from available sources, some data were not accessible. Many accessed survey reports were draft versions, and final reports may present different results (eg, after weighted analysis is conducted). Many reports did not describe basic survey elements such as representativeness, sample size, sampling methods, and eligibility criteria. Lack of such basic elements limited the establishment of consistent quality metrics or a composite scoring system. The categorization, a preliminary attempt to assess data availability and quality in combination, did not factor the consistency in sampling methods across years. Nor was the comparability of data across countries over time assessed. HIV prevalence is less sensitive than HIV incidence or mortality in gauging the impact of the HIV response. As incidence and viral suppression data become available, this categorization approach may be useful to understand the monitoring quality of these critical indicators. Moreover, trend analysis requires a minimum of 3 data points at the same SNU, with consistent application of probability sampling or approximate probability sampling methods. This definition yielded few SNUs for trend analysis, as the majority of the SNUs applied only nonprobability sampling methods. Last, the sample size selection of 100 as the threshold was arbitrary. Sample size considerations of each individual survey could not be reviewed for many of the available data.

Notwithstanding a slight decline in the availability of HIV prevalence data among SWs from 2001 to 2017, availability of data from the other reviewed populations increased during the same period. This increase is partly attributable to increased global financial investments and technical advances in implementing surveys among difficult-to-survey populations. Overall, however, the continued inadequacy of the most essential data is concerning. Regardless of the epidemic settings, basic epidemiologic data are essential for monitoring HIV responses and understanding progress toward the 95-95-95 goals. Ultimately, the success of the HIV response will hinge on our ability to ensure service coverage and quality in the last mile toward ending HIV as a public health threat by 2030. The global goal of ending HIV as an epidemic cannot be achieved without controlling the epidemic among KPs.

## Appendix

Multimedia Appendix 1 Country names.

Multimedia Appendix 2 Overall number of data points.

## Abbreviations

ANC: antenatal care
BBS: bio-behavioral surveys
FSW: female sex worker
IDU: injecting drug use
KP: key population
MSM: men who have sex with men
NGO: nongovernmental organizations
PWID: people who inject drugs
RDS: respondent-driven sampling
SNU: subnational unit
SW: sex worker
TG: transgender
TLS: time-location sampling
UNAIDS: United Nations Programme on HIV/AIDS
WHO: World Health Organization
