# Supplementary material for: Availability and Quality of Surveillance and Survey Data on HIV Prevalence Among Sex Workers, Men Who Have Sex With Men, People Who Inject Drugs, and Transgender Women in Low- and Middle-Income Countries: Review of Available Data (2001-2017)
Source: JMIR Public Health Surveill. 2020 Nov 17;6(4):e21688. doi: 10.2196/21688 (PMC7708087; doi:10.2196/21688)
Supplement: Multimedia Appendix 1 [file publichealth_v6i4e21688_app1.docx]

Multimedia Appendix 1 – Country Names

Afghanistan, Albania, Algeria, Angola, Armenia, Azerbaijan, Bangladesh, Belarus, Belize, Benin, Bhutan, Bolivia, Bosnia and Herzegovina, Botswana, Bulgaria, Burkina Faso, Burundi, Cabo Verde, Cambodia, Cameroon, Central Africa Republic, Chad, Columbia, Comoros, Republic of Congo, Democratic Republic of Congo, Costa Rica, Cote d’Ivoire, Cuba, Djibouti, Dominica, Dominica Republic, Ecuador, Egypt, El Salvador, Eritrea, Eswatini, Ethiopia, Gabon, Gambia, Georgia, Ghana, Grenada, Guatemala, Guinea, Guinea Bissau, Guyana, Haiti, Honduras, India, Indonesia, Iran, Jamaica, Kazakhstan, Kenya, Kiribati, Democratic People’s Republic of Korea, Kosovo, Kyrgyzstan, Laos, Lesotho, Liberia, Madagascar, Malawi, Malaysia, Maldives, Mali, Marshall Islands, Mauritania, Mauritius, Micronesia, Moldova, Mongolia, Morocco, Mozambique, Myanmar, Namibia, Nepal, Nicaragua, Niger, Nigeria, Pakistan, Palestine (West Bank and Gaza), Panama, Papua New Guinea, Paraguay, Peru, Philippines, Romania, Russia Federation, Rwanda, Samoa, Sao Tome and Principe, Senegal, Seychelles, Sierra Leone, Solomon Islands, Somalia, South Africa, South Sudan, Sri Lanka, St. Lucia, St. Vincent & Grenadines, Sudan, Suriname, Syria, Tajikistan, Tanzania, Thailand, Timor Leste, Togo, Tonga, Tunisia, Tuvalu, Uganda, Ukraine, Uzbekistan, Vanuatu, Viet Nam, Yemen, Zambia, Zanzibar, and Zimbabwe
