# Supplementary material for: Availability and Quality of Surveillance and Survey Data on HIV Prevalence Among Sex Workers, Men Who Have Sex With Men, People Who Inject Drugs, and Transgender Women in Low- and Middle-Income Countries: Review of Available Data (2001-2017)
Source: JMIR Public Health Surveill. 2020 Nov 17;6(4):e21688. doi: 10.2196/21688 (PMC7708087; doi:10.2196/21688)
Supplement: Multimedia Appendix 2 [file publichealth_v6i4e21688_app2.docx]

Multimedia Appendix 2 – Overall number of data points, those with reported sample size, those with sample size greater than 100, by key population over time*

|  | **2001** | **2002** | **2003** | **2004** | **2005** | **2006** | **2007** | **2008** | **2009** | **2010** | **2011** | **2012** | **2013** | **2014** | **2015** | **2016** | **2017** | **Total** | % |  |
| --- | --- | --- | --- | --- | --- | --- | --- | --- | --- | --- | --- | --- | --- | --- | --- | --- | --- | --- | --- | --- |
| **Sex workers** |  |  |  |  |  |  |  |  |  |  |  |  |  |  |  |  |  |  |  |  |
| Number of data points | 348 | 267 | 390 | 247 | 174 | 258 | 233 | 151 | 388 | 317 | 424 | 229 | 138 | 311 | 198 | 106 | 189 | 4,368 |  |  |
| With sample size available | 75 | 153 | 139 | 104 | 71 | 157 | 180 | 108 | 201 | 183 | 234 | 94 | 122 | 192 | 191 | 106 | 160 | 2,470 | 56·5% |  |
| With samples size > 100 | 50 | 109 | 84 | 71 | 53 | 123 | 139 | 84 | 144 | 164 | 202 | 83 | 96 | 141 | 135 | 91 | 117 | 1,886 | 76·4% |  |
| **Men who have sex with men** | | | | | | | | | | | | | | | | | | | | |
| Number of data points | 14 | 31 | 31 | 36 | 43 | 52 | 74 | 58 | 106 | 102 | 176 | 87 | 118 | 109 | 202 | 63 | 154 | 1,456 |  |  |
| With sample size available | 13 | 21 | 19 | 24 | 16 | 42 | 67 | 56 | 82 | 86 | 132 | 68 | 107 | 106 | 189 | 61 | 128 | 1,217 | 83·6% |  |
| With samples size > 100 | 11 | 18 | 18 | 21 | 11 | 38 | 63 | 48 | 72 | 67 | 112 | 50 | 100 | 81 | 156 | 32 | 104 | 1,002 | 82·3% |  |
| **People who inject drugs** | | | | | | | | | | | | | | | | | | | | |
| Number of data points | 92 | 105 | 88 | 140 | 113 | 178 | 176 | 134 | 198 | 111 | 215 | 79 | 85 | 121 | 131 | 88 | 157 | 2,211 |  |  |
| With sample size available | 14 | 21 | 44 | 39 | 27 | 81 | 114 | 77 | 101 | 47 | 99 | 55 | 69 | 78 | 125 | 82 | 121 | 1,194 | 54·0% |  |
| With samples size > 100 | 11 | 19 | 23 | 30 | 21 | 65 | 88 | 71 | 90 | 43 | 84 | 49 | 60 | 58 | 116 | 63 | 111 | 1,002 | 83·9% |  |
| **Transwomen** | | | | | | | | | | | | | | | | | | | | |
| Number of data points | 1 | 3 | 2 | 1 | 14 | 2 | 9 | 10 | 10 | 26 | 24 | 30 | 13 | 18 | 22 | 15 | 73 | 273 |  |  |
| With sample size available | 1 | 2 | 2 | 1 | 9 | 1 | 5 | 10 | 8 | 21 | 21 | 28 | 9 | 16 | 21 | 15 | 56 | 226 | 82·8% |  |
| With samples size > 100 | 0 | 1 | 1 | 1 | 8 | 1 | 4 | 8 | 8 | 5 | 18 | 13 | 7 | 7 | 10 | 5 | 32 | 129 | 57·1% |  |

*There were 11 countries with their total population less than 250 thousand, which were Dominica, Grenada, Kiribati, Marshall Islands, Samoa, Sao Tome and Principe, Seychelles, Saint Lucia, Saint Vincent and the Grenadines, Tonga, Tuvalu.
